# Impact of the COVID-19 pandemic on oral health and psychosocial factors

**DOI:** 10.1038/s41598-022-07907-9

**Published:** 2022-03-16

**Authors:** Antonio Ciardo, Marlinde M. Simon, Sarah K. Sonnenschein, Christopher Büsch, Ti-Sun Kim

**Affiliations:** 1 Section of Periodontology, Department of Conservative Dentistry, Clinic for Oral, Dental and Maxillofacial Diseases, Heidelberg University Hospital, Im Neuenheimer Feld 400, 69120 Heidelberg, Germany; 2 Institute of Medical Biometry, University of Heidelberg, Heidelberg, Germany

**Keywords:** Epidemiology, Dental public health, Public health, Quality of life, Psychology and behaviour, Oral manifestations, Anxiety, Depression

## Abstract

The objective of this study was to investigate oral health-related quality of life (OHRQoL) in times of the COVID-19 pandemic and to examine a possible association to psychosocial factors like psychological stress and symptoms of depression and anxiety disorders. Secondary research questions were whether people changed oral hygiene regimens during the COVID-19 pandemic and to what extent dental symptoms existed and developed compared to pre-pandemic. For this cross-sectional study a survey has been conceptualized to determine OHRQoL, stress, depression and anxiety and their specific confounders in a German cohort. Validated questionnaires as OHIP-G14, PHQ-Stress and PHQ-4 have been implemented. Altogether 1178 participants completed the survey between May and August 2020. The overall OHIP-G14 sum score of 4.8 ± 7.5 indicated good OHRQoL. 21% of the participants (n = 248) reported toothache, 23% (n = 270) mucosal problems, 31% (n = 356) hypersensitivity of the teeth and 27% (n = 305) myofacial pain. The PHQ-Stress score (4.5 ± 3.5) demonstrated a mild severity of stress. Depression and anxiety level has been mild to moderate (PHQ-4 score: 2.4 ± 2.6). 38% of the participants stated subjectively greater emotional burden compared to pre-pandemic. Statistically significant differences exist for OHRQoL, stress, anxiety and depression levels between participants with greater, equal or less emotional burden compared to pre-pandemic. COVID-19 history and aggravated levels of depression, anxiety, and stress seem to associate with lower OHRQoL. Psychosocial consequences during pandemic times and their association to oral health should be further investigated.

## Introduction

Coronavirus disease 2019 (COVID-19) is a respiratory disease caused by the virus SARS-CoV-2. It was declared a pandemic by the World Health Organization in March 2020. The course of the disease is diverse and varies. In addition to asymptomatic infections, mild to moderate courses were observed, as well as severe progressions with pneumonia up to lung failure and death^1^. In order to contain the rapidly increasing number of infections and to prevent an overload of the health system, public life in Germany was severely restricted from March 2020. Hence, people were asked to stay at home and adhere to contact restrictions. Numerous shops and services had to close temporarily. In the meantime, outpatient dental care had also been reduced to a minimum. For children, classroom-teaching in schools and child care in day-care centers were temporarily cancelled. This led to a change of working conditions (home office, furlough, terminations, etc.) and social situations (e.g. family, friends)^2,3^. Due to decreasing incidences, public life was gradually allowed to resume again as of May 2020, but with continuing substantial restrictions. Effects of the COVID-19 pandemic and its countermeasures on the perception of psychological stress and mental health or on the intensification of already existing mental conditions were shown^4,5^.

The effect of psychological stress and mental conditions such as depression and anxiety disorders on the course of oral conditions as e.g. periodontitis or functional conditions and vice versa has not been conclusively established, but several studies show an association^6^. On the one hand, stress can influence the immune response and is generally associated with inflammation. On the other hand, health-related behavior as preventive oral hygiene can be negatively influenced by psychosocial factors, which in return can lead to increased infection or may aggravate existing inflammation^6–12^.

Oral health or disease in general, can be assessed objectively and clinically based, while neglecting the impact on patient’s quality of life^13,14^. A more patient-reported but subjective outcome is the concept of oral health-related quality of life (OHRQoL). Consequently, this measure cannot reveal the clinical oral status, but the individual’s perception of oral health and its impact on life^13^. It has to be interpreted as a multidimensional concept involving biopsychosocial aspects related to oral health^13^. It can be influenced by cultural context and age^13^. Instruments as the most widely used Oral Health Impact Profile (OHIP) capture the patient-perceived impact and make OHRQoL measurable^13,14^.

The objective of this study was to examine how adults in Germany rate their OHRQoL in times of the COVID-19 pandemic and whether an association to perceived psychosocial stress and symptoms of depressive disorders and generalized anxiety exist. Secondary research questions were whether people changed oral hygiene regimens during the COVID-19 pandemic and to what extent oral symptoms as toothache, mucosal pain, dental hypersensitivity or myofacial pain existed and developed compared to pre-pandemic.

## Methods

### Study design

This questionnaire-based cross-sectional study was approved by the ethics committee of the Medical Faculty of Heidelberg (# S-303/2020) and is in accordance with the ethical standards of the 1964 Helsinki Declaration and its later amendments or comparable ethical standards. The trial was registered at the US National Institute of Health (ClinicalTrials.gov, # NCT04381273). This observational study is being reported using the STROBE statement for cross-sectional studies^15^.

### Survey development

For this study, a digital questionnaire consisting of validated items was conceptualized to determine oral health and psychosocial factors and queries experiences in times of the COVID-19 pandemic. Hence, the questionnaire is conducted of sub-questionnaires and free questions (total: 27 questions). To query oral-health related and also sociodemographic data, the seven-item questionnaire from the German Society for Periodontology (DG PARO) “the periodontitis risk score” was integrated. The age range was slightly modified in order to include all ages from 18 years onwards^16^. The score ranges from 0 points indicating lowest periodontitis risk up to 20 points indicating very high periodontitis risk. A question was also added asking whether toothache, mucosal pain, dental hypersensitivity or myofacial pain were perceived symptoms and whether they were felt the same, stronger or weaker compared to before the pandemic. OHRQoL was assessed by the German version of the 14-item Oral Health Impact Profile (OHIP-G14). It consists of 14 questions which can be grouped into seven distinct sub-domains: (1) functional limitation (items 1 and 2), (2) handicap (items 3 and 10), (3) psychological disability (items 4 and 11), (4) psychological discomfort (items 5 and 14), (5) physical disability (items 6 and 12), (6) physical pain (items 7 and 13), and (7) social disability (items 8 and 9). The answers are recorded on a Likert scale with values ranging from 0 to 4 coded as 0 “never”, 1 “hardly ever”, 2 “occasionally”, 3 “fairly often”, or 4 “very often”. The OHIP-G14 sum score can range from 0 to 56 with a higher score indicating a poorer OHRQoL^17^. To determine psychosocial stress during the COVID-19 pandemic, the validated ten-item PHQ-Stress module (Patient Health Questionnaire) was chosen. The psychosocial impairment caused by the queried factors can be assessed from 0 “not impaired”, over 1 “slightly impaired” to 2 “severely impaired”. Score values of 0–4 can be viewed as minimally pronounced psychosocial stress factors. Total values of 5–9 represent mild, 10–14 moderate and 15–20 severe stress manifestation^18^. As a validated screening instrument for depressive disorders and generalized anxiety, the four-item PHQ-4 was included. The frequency of complaints inquired can be evaluated on a scale from 0 “not at all” to 3 “almost every day”. Total values can range from 0–12. A PHQ-4 score of 0–2 designates none-to-minimal, 3–5 mild, 6–8 moderate and 9–12 severe level of depression and anxiety^18,19^. Questions were added on the subjective assessment of the extent of pandemic-related restrictions in life (none/little/strong), as well as the extent to which the pandemic had an influence on work and family/social life (none/little/strong). An additional question was implemented on subjectively perceived emotional burden during the COVID-19 pandemic compared to before the pandemic (greater/equal/less) to also match these self-assessments in relation to the more objectified scores of the psychosocial sub-questionnaires as the PHQ-Stress and PHQ-4. In order to take known confounders for oral health and psychosocial factors into account, questions about the oral hygiene behavior, systemic diseases, body weight and height (to calculate the body mass index (BMI)) as well as the aforementioned demographic and socioeconomic questions regarding age, sex, smoking educational and employment status were added.

### Recruitment & investigation

The questionnaire was created using a “free access” for scientific research at “umfrageonline.com” (enuvo GmbH, Zurich, Switzerland) and was available online for twelve weeks from May 16th, 2020 to August 8th, 2020. Answering the questionnaire took about 5–10 min. Because the survey was anonymous and voluntary, and the purpose of the study was described at the beginning of the questionnaire, no additional informed consent was required, as approved by the ethics committee of the Medical Faculty of Heidelberg (# S-303/2020). The digital link was initially distributed to patients at the Clinic of Conservative Dentistry at Heidelberg University Hospital and published on a flyer. Passing on the link e.g. through social media was anticipated with the intention of reaching as many people of all ages as possible across Germany. Additionally, a printed version of the questionnaire was made available for people without internet access, reaching especially local respondents. Additional samples were distributed by some study participants. In order to fit the inclusion criteria participants had to be over 18 years of age, have a place of residence in Germany and give consent to participate in the study.

### Sample size & statistical analysis

Due to the nature of the survey, no formal sample size calculation was performed. Nevertheless, a power calculation using a Kruskal–Wallis one-way ANOVA in a study population of 1097 (365.7 per group) with mean OHIP-G14 sum score of 7.74, 3.05 and 3.80 for the three levels of emotional burden (greater/equal/less compared to pre-pandemic), respectively, yields a power of 100% using a type-I-error of 5%. Power calculation was performed by the software PASS v 16.0.3.

Demographics and general data were described using descriptive measures. Continuous variables were described using the number of non-missing values, mean and standard deviation. For binary or categorical variables absolute and relative frequencies were provided.

To investigate OHRQoL (primary objective), data from the OHIP-G14 was collected and analyzed using Kruskal–Wallis tests between participants with a greater, equal or lower emotional burden compared to pre-pandemic for each of the seven sub-domains and the sum score of the OHIP-G14. Non-parametric tests were chosen because of the skewed allocation ratio and the not normally distributed data. All missing values were excluded for statistical testing.

The influence of psychosocial factors and confounders on OHRQoL was examined using a linear regression model with stepwise (bidirectional elimination) variable selection based on Akaike information criterion. As dependent variable, the OHIP-G14 sum score and as independent variables, the following were used (variables marked with a * were considered clinically relevant and hence were enforced to be included in the final model and only the variables not marked with a * were used in the variable selection process): “PHQ-Stress sum score”*, “PHQ-4 sum score”*, “sex” (male/female)*, “age group” (18–39/40–59/60–100)*, “systemic diseases” (yes/no), “COVID-19 history” (yes/no), “employment status” (employed/self-employed/unemployed/student/in training/retired), “restrictions due to the COVID-19-pandemic” (strong/little/none), “BMI”, “periodontitis-history” (yes/no), “educational status” (≤ 10 years/ > 10 years of school), “interdental cleaning” (yes/no), “dentist visit frequency” (2/1/ < 1 per year), “tooth brushing frequency” (≤ 1/ ≥ 2 per day) and “emotional burden due to the COVID-19-pandemic compared to pre-pandemic” (greater/equal/less).

The secondary objective whether people changed oral hygiene regimens during the COVID-19 pandemic was analyzed using descriptive measures as described above and additionally Chi-squared tests between patients with greater, equal and less emotional burden compared to pre-pandemic.

Furthermore, if one item of a sub-questionnaire was missing, the whole sub-questionnaire was defined missing for this participant and excluded for statistical testing. In addition, due to the exploratory character of the survey, p-values can only be interpreted descriptively, so that no formal adjustment was made for multiple testing. P-values smaller than 0.05 were considered to be statistically significant. Statistical analyses were conducted using the statistic software R (version 4.0.2, R Core Team, Auckland, New Zealand) and were carried out at the Institute for Medical Biometry at Heidelberg University Hospital.

## Results

### Study population

A total of 1178 participants (426 men (36%), 747 women (64%)) took part in the survey, n = 795 (67%) digitally and n = 383 (33%) analogously. All age groups were represented, but not evenly distributed. The participants included residents of all 16 federal states, but with local differences in distribution. In particular, there were many participants from Baden-Württemberg (n = 488, 44%) and Hesse (n = 271, 25%). In Table 1, further demographic information is listed and additionally divided into study participants with a greater (n = 429), equal (n = 594) or lower (n = 134) emotional burden compared to pre-pandemic.

**Table 1:** Demographic and general data of the study cohort.

Variables	Total (n = 1178)	Greater emotional burden compared to pre-pandemic (n = 429)	Equal emotional burden compared to pre-pandemic (n = 594)	Less emotional burden compared to pre-pandemic (n = 134)	Emotional burden compared to pre-pandemic data missing (n = 21)
**Age group**					
18–29 years	215 (18%)	83 (19%)	99 (17%)	30 (22%)	3 (17%)
30–39 years	192 (16%)	83 (19%)	86 (15%)	19 (14%)	4 (22%)
40–49 years	129 (11%)	57 (13%)	57 (10%)	14 (10%)	1 (6%)
50–59 years	236 (20%)	109 (25%)	109 (18%)	18 (13%)	0 (0%)
60–69 years	194 (17%)	64 (15%)	106 (18%)	21 (16%)	3 (17%)
70–79 years	134 (11%)	18 (4%)	94 (16%)	21 (16%)	1 (6%)
80–89 years	67 (6%)	13 (3%)	39 (7%)	10 (7%)	5 (28%)
90–99 years	3 (0%)	0 (0%)	2 (0%)	1 (1%)	0 (0%)
≥ 100 years	2 (0%)	1 (0%)	0 (0%)	0 (0%)	1 (6%)
Missing	6	1	2	0	3
**Sex**					
Male	426 (36%)	106 (25%)	251 (42%)	58 (44%)	11 (58%)
Female	747 (64%)	322 (75%)	342 (58%)	75 (56%)	8 (42%)
Missing	5	1	1	1	2
**Smoking**					
Never smoker	623 (53%)	221 (52%)	322 (54%)	66 (50%)	14 (74%)
Former smoker	363 (31%)	130 (30%)	190 (32%)	39 (29%)	4 (21%)
Active smoker, < 10 cigarettes per day	84 (7%)	38 (9%)	35 (6%)	11 (8%)	0 (0%)
Active smoker, ≥ 10 cigarettes per day	104 (9%)	40 (9%)	46 (8%)	17 (13%)	1 (5%)
Missing	4	0	1	1	2
**Body mass index (BMI)**					
n (missing)	1132 (46; 4%)	416 (13; 3%)	577 (17; 3%)	128 (6; 4%)	11 (10; 48%)
BMI ± SD	25.0 ± 4.8	25.0 ± 5.3	25.0 ± 4.5	25.0 ± 4.6	25.0 ± 3.3
**Educational status**					
≤ 10 years of school	352 (30%)	118 (28%)	183 (31%)	40 (31%)	11 (61%)
> 10 years of school	817 (70%)	311 (72%)	408 (69%)	91 (69%)	7 (39%)
Missing	9	0	3	3	3
**Employment status**					
Employed	538 (47%)	228 (54%)	261 (44%)	47 (36%)	2 (18%)
Self-employed	138 (12%)	68 (16%)	55 (9%)	15 (11%)	0 (0%)
Unemployed	33 (3%)	11 (3%)	15 (3%)	7 (5%)	0 (0%)
Student/In Training	126 (11%)	50 (12%)	58 (10%)	18 (14%)	0 (0%)
Retired	320 (28%)	66 (16%)	200 (34%)	45 (34%)	9 (82%)
Missing	23	6	5	2	10
**Systemic diseases**					
Lung disease (COPD, Asthma, etc.)	93 (8%)	46 (11%)	37 (6%)	10 (7%)	0 (0%)
Cardiovascular disease	161 (14%)	54 (13%)	87 (15%)	17 (13%)	3 (1%)
Diabetes mellitus	63 (5%)	19 (44%)	29 (5%)	12 (9%)	3 (1%)
Other systemic diseases	215 (18%)	92 (21%)	96 (16%)	26 (19%)	1 (0%)
No systemic diseases	698 (59%)	243 (57%)	373 (63%)	78 (58%)	4 (2%)
**Federal state**					
Baden-Württemberg	488 (44%)	153 (37%)	263 (47%)	66 (54%)	6 (60%)
Bavaria	55 (5%)	33 (8%)	17 (3%)	5 (4%)	0 (0%)
Berlin	16 (1%)	8 (2%)	7 (1%)	1 (1%)	0 (0%)
Brandenburg	3 (0%)	2 (0%)	1 (0%)	0 (0%)	0 (0%)
Bremen	1 (0%)	1 (0%)	0 (0%)	0 (0%)	0 (0%)
Hamburg	4 (0%)	2 (0%)	2 (0%)	0 (0%)	0 (0%)
Hesse	271 (25%)	84 (20%)	155 (28%)	29 (24%)	3 (30%)
Mecklenburg-Vorpommern	2 (0%)	1 (0%)	1 (0%)	0 (0%)	0 (0%)
Lower Saxony	41 (4%)	26 (6%)	14 (2%)	1 (1%)	0 (0%)
North Rhine-Westphalia	105 (10%)	49 (12%)	47 (8%)	9 (7%)	0 (0%)
Rhineland-Palatinate	83 (8%)	33 (8%)	41 (7%)	8 (7%)	1 (10%)
Saarland	3 (0%)	3 (1%)	0 (0%)	0 (0%)	0 (0%)
Saxony	10 (1%)	6 (1%)	4 (1%)	0 (0%)	0 (0%)
Saxony-Anhalt	5 (0%)	3 (1%)	2 (0%)	0 (0%)	0 (0%)
Schleswig–Holstein	2 (0%)	1 (0%)	1 (0%)	0 (0%)	0 (0%)
Thuringia	16 (1%)	8 (2%)	5 (1%)	3 (2%)	0 (0%)
Missing	73	16	34	12	11
**COVID-19 anamnesis**					
No COVID-19 history	1136 (98%)	418 (98%)	583 (98%)	130 (99%)	5 (100%)
Confirmed COVID-19 history without hospitalization	14 (1%)	4 (1%)	9 (2%)	1 (1%)	0 (0%)
Confirmed COVID-19 history and hospitalization	5 (0%)	5 (1%)	0 (0%)	0 (0%)	0 (0%)
Missing	23	2	2	3	16
**Pandemic-related restrictions in life**					
Strong restrictions	295 (26%)	192 (45%)	83 (14%)	18 (14%)	2 (18%)
Little restrictions	710 (62%)	218 (51%)	401 (68%)	84 (65%)	7 (64%)
No restrictions	146 (13%)	14 (3%)	102 (17%)	28 (22%)	2 (18%)
Missing	27	5	8	4	10
**Pandemic-related influence on work**					
Strong influence	431 (41%)	238 (59%)	153 (29%)	40 (34%)	0 (0%)
Little influence	319 (30%)	105 (26%)	179 (34%)	34 (29%)	1 (17%)
No influence	308 (29%)	59 (15%)	200 (38%)	44 (37%)	5 (83%)
Missing	120	27	62	16	15
**Pandemic-related influence on family/social life**					
Strong influence	519 (46%)	291 (69%)	185 (32%)	42 (33%)	1 (12%)
Little influence	494 (43%)	122 (29%)	306 (53%)	60 (47%)	6 (75%)
No influence	125 (11%)	8 (2%)	91 (16%)	25 (20%)	1 (12%)
Missing	40	8	12	7	13

*n* number of participants, *COVID-19* coronavirus disease 2019, *SD* standard deviation.

Of all respondents, 295 (26%) stated that their daily life was severely restricted by the COVID-19 pandemic, 710 (62%) stated that they were only slightly restricted and 146 (13%) evaluated no restrictions. The COVID-19 pandemic exerted a strong influence on everyday work for n = 431 (41%), a minor influence for n = 319 (30%) and no influence for n = 308 (29%). With regard to family and social environment, n = 519 (46%) felt a strong influence, n = 494 (43%) a slight and n = 125 (11%) no influence.

### Oral health-related parameters

The first seven questions of the questionnaire included validated questions of the DG PARO periodontitis risk score. Overall (n = 1149), a mean value of 6.9 ± 4.0 was given. 421 participants with subjectively greater emotional burden compared to pre-pandemic reported a periodontitis risk score of 6.2 ± 3.6 compared to 587 participants with equal emotional burden and a score of 7.4 ± 4.1 and 124 with less emotional burden and a score of 7.1 ± 4.3, respectively (p < 0.001^KW^). Perception of periodontitis and dentist visit frequency are shown in Table 2. With regard to oral hygiene measures, 899 participants (77%) reported that they brushed their teeth at least twice per day, 257 (22%) once per day and 15 (1%) less than once per day. There was no statistically significant difference between the three groups on this topic (p = 0.270^chi2^), in contrast to interdental cleaning (p = 0.032^chi2^). Overall, n = 512 (44%) used interdental brushes, n = 317 (27%) used floss, n = 82 (7%) other aids and n = 259 (22%) did not use any aids for interdental care. In total, 1083 (93%) of those surveyed had not changed toothbrushing frequency during the pandemic. Participants with greater emotional burden stated more frequently that they had noticed worsening of toothache (6% vs. 3% vs. 3%, p = 0.006^chi2^), mucosal pain (7% vs. 3% vs. 4%, p = 0.029^chi2^), hypersensitivity (9% vs. 3% vs. 4%, p < 0.001^chi2^) and myofacial pain (13% vs. 3% vs. 2%, p < 0.001^chi2^) during the pandemic compared to those with equal or less emotional burden compared to pre-pandemic.

**Table 2:** Oral health-related parameters.

Variables	Total (n = 1178)	Greater emotional burden compared to pre-pandemic (n = 429)	Equal emotional burden compared to pre-pandemic (n = 594)	Less emotional burden compared to pre-pandemic (n = 134)	Emotional burden compared to pre-pandemic data missing (n = 21)	p-Value
**Bleeding of the gums**						
No	787 (67%)	271 (63%)	410 (69%)	92 (70%)	14 (74%)	
Sometimes	357 (30%)	143 (33%)	171 (29%)	38 (29%)	5 (26%)	
Often	27 (2%)	14 (3%)	11 (2%)	2 (2%)	0 (0%)	
Missing	7	1	2	2	2	
**Tooth mobility**						
No	1052 (91%)	382 (91%)	539 (91%)	115 (88%)	16 (84%)	
Yes	110 (9%)	40 (9%)	52 (9%)	15 (12%)	3 (16%)	
Missing	16	7	3	4	2	
**Periodontitis risk score**						** < 0.001**^**KW**^
n	1149	421	587	124	17	
Missing	29 (2%)	8 (2%)	7 (1%)	10 (7%)	4 (19%)	
Mean	6.9	6.2	7.4	7.1	8.2	
SD	4.0	3.6	4.1	4.3	5.0	
Median	7	6	8	8	9	
Q1–Q3	3–10	3–9	4–11	3–11	4–12	
Min–Max	0–17	0–16	0–17	0–15	2–16	
**Periodontitis anamnesis**						
Yes, treated	361 (31%)	109 (26%)	199 (34%)	49 (37%)	4 (21%)	
Yes, not treated	26 (2%)	9 (2%)	14 (2%)	3 (2%)	0 (0%)	
No	659 (56%)	256 (61%)	324 (55%)	66 (50%)	10 (53%)	
Unknown	122 (10%)	48 (11%)	55 (9%)	14 (11%)	5 (26%)	
Missing	10	4	2	2	2	
**Dental status**						
I have natural teeth	1031 (88%)	386 (90%)	516 (87%)	116 (87%)	13 (62%)	
I am edentulous	11 (1%)	3 (1%)	6 (1%)	1 (1%)	1 (5%)	
I have implants	264 (22%)	92 (21%)	140 (24%)	27 (20%)	5 (24%)	
I wear dentures	113 (10%)	32 (7%)	61 (10%)	17 (13%)	3 (14%)	
**Dentist visit frequency**						0.671^chi2^
2/year	658 (56%)	252 (59%)	322 (54%)	77 (58%)	7 (39%)	
1/year	380 (32%)	128 (30%)	201 (34%)	42 (32%)	9 (50%)	
< 1/year	133 (11%)	48 (11%)	69 (12%)	14 (11%)	2 (11%)	
Missing	7	1	2	1	3	
**Tooth brushing frequency**						0.270^chi2^
≥ 2/day	899 (77%)	333 (78%)	456 (77%)	96 (72%)	14 (78%)	
1/day	257 (22%)	92 (21%)	128 (22%)	33 (25%)	4 (22%)	
< 1/day	15 (1%)	3 (1%)	8 (1%)	4 (3%)	0 (0%)	
Missing	7	1	2	1	3	
**Interdental cleaning**						**0.032**^**chi2**^
Yes, with interdental brushes	512 (44%)	165 (39%)	282 (48%)	56 (42%)	9 (50%)	
Yes, with dental floss	317 (27%)	136 (32%)	143 (24%)	34 (26%)	4 (22%)	
Yes, with other tools	82 (7%)	26 (6%)	41 (7%)	14 (11%)	1 (6%)	
No	259 (22%)	101 (24%)	126 (21%)	28 (21%)	4 (22%)	
Missing	8	1	2	2	3	
**Oral hygiene frequency during COVID-19 pandemic compared to before**						**0.001**^**chi2**^
Less often	34 (3%)	23 (5%)	7 (1%)	3 (2%)	1 (6%)	
Unchanged	1083 (93%)	384 (90%)	562 (95%)	121 (91%)	16 (94%)	
More often	53 (5%)	20 (5%)	24 (4%)	9 (7%)	0 (0%)	
Missing	8	2	1	1	4	
**Toothache**						**0.006**^**chi2**^
Worse than pre-pandemic	46 (4%)	25 (6%)	17 (3%)	4 (3%)	0 (0%)	
Equivalent to pre-pandemic	200 (17%)	90 (21%)	93 (16%)	17 (13%)	0 (0%)	
Better than pre-pandemic	2 (0%)	0 (0%)	1 (0%)	1 (1%)	0 (0%)	
None	904 (78%)	308 (73%)	479 (81%)	111 (83%)	6 (100%)	
Missing	26	6	4	1	15	
**Mucosal symptoms**						**0.029**^**chi2**^
Worse than pre-pandemic	55 (5%)	30 (7%)	20 (3%)	5 (4%)	0 (0%)	
Equivalent to pre-pandemic	212 (18%)	89 (21%)	104 (18%)	19 (14%)	0 (0%)	
Better than pre-pandemic	3 (0%)	2 (0%)	1 (0%)	0 (0%)	0 (0%)	
None	882 (77%)	302 (71%)	464 (79%)	110 (82%)	6 (100%)	
Missing	26	6	5	0	15	
**Dental hypersensitivity**						** < 0.001**^**chi2**^
Worse than pre-pandemic	58 (5%)	38 (9%)	15 (3%)	5 (4%)	0 (0%)	
Equivalent to pre-pandemic	291 (25%)	120 (29%)	148 (25%)	23 (17%)	0 (0%)	
Better than pre-pandemic	7 (1%)	2 (0%)	4 (1%)	1 (1%)	0 (0%)	
None	796 (69%)	261 (62%)	424 (72%)	105 (78%)	6 (100%)	
Missing	26	8	3	0	15	
**Myofacial pain**						** < 0.001**^**chi2**^
Worse than pre-pandemic	75 (7%)	55 (13%)	17 (3%)	3 (2%)	0 (0%)	
Equivalent to pre-pandemic	223 (19%)	89 (21%)	110 (19%)	24 (18%)	0 (0%)	
Better than pre-pandemic	7 (1%)	2 (0%)	2 (0%)	3 (2%)	0 (0%)	
None	846 (74%)	276 (65%)	460 (78%)	104 (78%)	6 (100%)	
Missing	27	7	5	0	15	

Significant values are in bold.

All missing values were excluded for statistical testing.

*n* number of participants, *COVID-19* coronavirus disease 2019, *SD* standard deviation, *Q* Quartile, *Min* Minimum, *Max* Maximum, *MWU* Mann–Whitney U test, *chi2* chi-squared test, *KW* Kruskal–Wallis one-way ANOVA.

### Psychosocial factors

Overall, the respondents gave a total sum score of 4.5 ± 3.5 for the validated questions of the PHQ-Stress module. Regarding depression and anxiety, the participants gave a total of 2.4 ± 2.6 points in the PHQ-4 sum score (Table 3). Concerning whether people were emotionally strained differently during the COVID-19 pandemic compared to before, 38% of all respondents (n = 429) stated a much greater or greater burden, while 12% (n = 134) stated less or much less burden. When dividing the study cohort in participants with greater, equal and less emotional burden compared to pre-pandemic, the PHQ-Stress sum score differed statistically significantly (p < 0.001^KW^) by total values of 6.6 ± 3.4, 3.2 ± 2.7 and 3.4 ± 3.1, respectively. Also, the PHQ-4 sum score differed in this group comparison statistically significantly (p < 0.001^KW^) by 4.0 ± 2.8, 1.4 ± 1.7 and 1.4 ± 1.8, respectively.

**Table 3:** Psychosocial factors.

Variables	Total (n = 1178)	Greater emotional burden compared to pre-pandemic (n = 429)	Equal emotional burden compared to pre-pandemic (n = 594)	Less emotional burden compared to pre-pandemic (n = 134)	Emotional burden compared to pre-pandemic data missing (n = 21)	p-Value
**PHQ-stress**						** < 0.001**^**KW**^
n	1043	395	530	115	3	
Missing	135 (11%)	34 (8%)	64 (11%)	19 (14%)	18 (86%)	
Mean	4.5	6.6	3.2	3.4	2.7	
SD	3.5	3.4	2.7	3.1	3.8	
Median	4	6	3	3	1	
Q1–Q3	2–7	4–9	1–5	1–4	0–7	
Min–Max	0–20	0–19	0–13	0–20	0–7	
**PHQ-4**						** < 0.001**^**KW**^
n	1141	420	584	129	8	
Missing	37 (3%)	9 (2%)	10 (2%)	5 (4%)	13 (62%)	
Mean	2.4	4	1.4	1.4	1.8	
SD	2.6	2.8	1.7	1.8	2.5	
Median	2	4	1	1	0.5	
Q1–Q3	0–4	2–5	0–2	0–2	0–3	
Min–Max	0–12	0–12	0–12	0–12	0–7	
**Emotional burden compared to pre-pandemic**						
Much greater	64 (6%)	64 (15%)	0 (0%)	0 (0%)		
Greater	365 (32%)	365 (85%)	0 (0%)	0 (0%)		
Equal	594 (51%)	0 (0%)	594 (100%)	0 (0%)		
Less	100 (9%)	0 (0%)	0 (0%)	100 (75%)		
Much less	34 (3%)	0 (0%)	0 (0%)	34 (25%)		
Missing	21	0	0	0	21	

Significant values are in bold.

All missing values were excluded for statistical testing.

*n* number of participants, *COVID-19* coronavirus disease 2019, *SD* standard deviation,* Q* Quartile, *Min* Minimum, *Max* Maximum, *KW* Kruskal–Wallis one-way ANOVA.

### OHRQoL

On average, 1103 study participants reached an OHIP-G14 sum score of 4.8 ± 7.5 (Table 4). When dividing the study cohort in participants with greater, equal and less emotional burden compared to pre-pandemic, the overall OHIP-G14 sum score was 7.7 ± 9.4, 3.1 ± 5.1 and 3.8 ± 7.1, respectively, with statistically significant difference (p < 0.001^KW^). These differences are present in all the seven OHIP-G14 sub-domains.

**Table 4:** Oral health-related quality of life.

Variables	Total (n = 1178)	Greater emotional burden compared to pre-pandemic (n = 429)	Equal emotional burden compared to pre-pandemic (n = 594)	Less emotional burden compared to pre-pandemic (n = 134)	Emotional burden compared to pre-pandemic data missing (n = 21)	p-Value
**OHIP-G14—total sum score**						** < 0.001**^**KW**^
n	1103	402	567	128	6	
Missing	75 (6%)	27 (6%)	27 (5%)	6 (4%)	15 (71%)	
Mean	4.8	7.7	3.1	3.8	2	
SD	7.5	9.4	5.1	7.1	2.6	
Median	2	4	1	1.5	1.5	
Q1–Q3	0–6	1–11	0–4	0–4	0–2	
Min–Max	0–53	0–53	0–37	0–48	0–7	
**OHIP-G14—sub-domain 1 (functional limitation)**						**0.007**^**KW**^
n	1145	417	589	132	7	
Missing	33 (3%)	12 (3%)	5 (1%)	2 (1%)	14 (67%)	
Mean	0.28	0.37	0.22	0.27	0.14	
SD	0.91	1	0.79	1	0.38	
Median	0	0	0	0	0	
Q1–Q3	0–0	0–0	0–0	0–0	0–0	
Min–Max	0–8	0–8	0–6	0–8	0–1	
**OHIP-G14—sub-domain 2 (handicap)**						** < 0.001**^**KW**^
n	1143	417	586	134	6	
Missing	35 (3%)	12 (3%)	8 (1%)	0 (0%)	15 (71%)	
Mean	0.88	1.5	0.48	0.6	0.67	
SD	1.4	1.8	0.92	1.3	0.82	
Median	0	1	0	0	0.5	
Q1–Q3	0–1	0–2	0–1	0–1	0–1	
Min–Max	0–8	0–8	0–8	0–8	0–2	
**OHIP-G14—sub-domain 3 (psychological disability)**						** < 0.001**^**KW**^
n	1137	415	583	133	6	
Missing	41 (3%)	14 (3%)	11 (2%)	1 (1%)	15 (71%)	
Mean	1	1.7	0.6	0.65	0.5	
SD	1.5	1.8	1	1.2	0.55	
Median	0	1	0	0	0.5	
Q1–Q3	0–2	0–3	0–1	0–1	0–1	
Min–Max	0–8	0–8	0–6	0–8	0–1	
**OHIP-G14—sub-domain 4 (psychological discomfort)**						** < 0.001**^**KW**^
n	1144	418	587	133	6	
Missing	34 (3%)	11 (3%)	7 (1%)	1 (1%)	15 (71%)	
Mean	0.77	1.2	0.53	0.59	0.17	
SD	1.5	1.9	1.1	1.1	0.41	
Median	0	0	0	0	0	
Q1–Q3	0–1	0–2	0–1	0–1	0–0	
Min–Max	0–8	0–8	0–6	0–6	0–1	
**OHIP-G14—sub-domain 5 (physical disability)**						** < 0.001**^**KW**^
n	1146	420	588	132	6	
Missing	32 (3%)	9 (2%)	6 (1%)	2 (1%)	15 (71%)	
Mean	0.48	0.79	0.27	0.47	0	
SD	1.2	1.5	0.81	1.2	0	
Median	0	0	0	0	0	
Q1–Q3	0–0	0–1	0–0	0–0	0–0	
Min–Max	0–8	0–8	0–7	0–7	0–0	
**OHIP-G14—sub-domain 6 (physical pain)**						** < 0.001**^**KW**^
n	1147	419	589	133	6	
Missing	31 (3%)	10 (2%)	5 (1%)	1 (1%)	15 (71%)	
Mean	0.6	0.87	0.43	0.5	0	
SD	1.2	1.4	1	1.1	0	
Median	0	0	0	0	0	
Q1–Q3	0–1	0–1	0–0	0–0	0–0	
Min–Max	0–8	0–8	0–7	0–6	0–0	
**OHIP-G14—sub-domain 7 (social disability)**						** < 0.001**^**KW**^
n	1147	420	587	134	6	
Missing	31 (3%)	9 (2%)	7 (1%)	0 (0%)	15 (71%)	
Mean	0.94	1.5	0.58	0.81	0.5	
SD	1.6	2	1.2	1.6	1.2	
Median	0	0	0	0	0	
Q1–Q3	0–1	0–3	0–1	0–1	0–0	
Min–Max	0–8	0–8	0–8	0–8	0–3	

Significant values are in bold.

All missing values were excluded for statistical testing.

*n* number of participants,* COVID-19* coronavirus disease 2019, *OHIP* Oral Health Impact Profile, *SD* standard deviation, *Q* Quartile, *Min* Minimum, *Max* Maximum, *KW* Kruskal–Wallis one-way ANOVA.

### Influencing factors on OHRQoL

The variable “periodontitis-history” was not included in the stepwise linear regression due to many missing values. The factors “BMI”, “educational status”, “interdental cleaning”, “dentist visit frequency”, “tooth brushing frequency” and “emotional burden due to the COVID-19-pandemic compared to pre-pandemic” cannot be found in the final output of the regression (Table 5) due to the elimination process of the performed variable selection (bidirectional elimination) based on Akaike information criterion.

**Table 5:** Stepwise linear regression result for modeling OHIP-G14 sum score with fixed effects (n = 928).

Variable	Estimate	Lower 95% CI	Upper 95% CI	p-value
Intercept	− 1.625	− 2.614	− 0.636	**0.001**
PHQ-Stress sum score	0.585	0.440	0.730	** < 0.001**
PHQ-4 sum score	0.973	0.778	1.169	** < 0.001**
Sex: female	− 0.240	− 1.035	0.555	0.554
Age group: 40–59 years	0.848	− 0.059	1.755	0.067
Age group: 60–100 years	1.706	0.707	2.704	** < 0.001**
COVID-19 history: yes	12.050	9.078	15.022	** < 0.001**
Systemic diseases: yes	0.890	0.064	1.716	**0.035**
Pandemic-related restrictions in life: strong	0.287	− 1.134	1.707	0.692
Pandemic-related restrictions in life: little	− 0.617	− 1.817	0.583	0.313

Significant values are in bold.

*CI* confidence interval, *OHIP* Oral Health Impact Profile, *PHQ* Patient Health Questionnaire, *COVID-19* coronavirus disease 2019.

**Figure 1:**
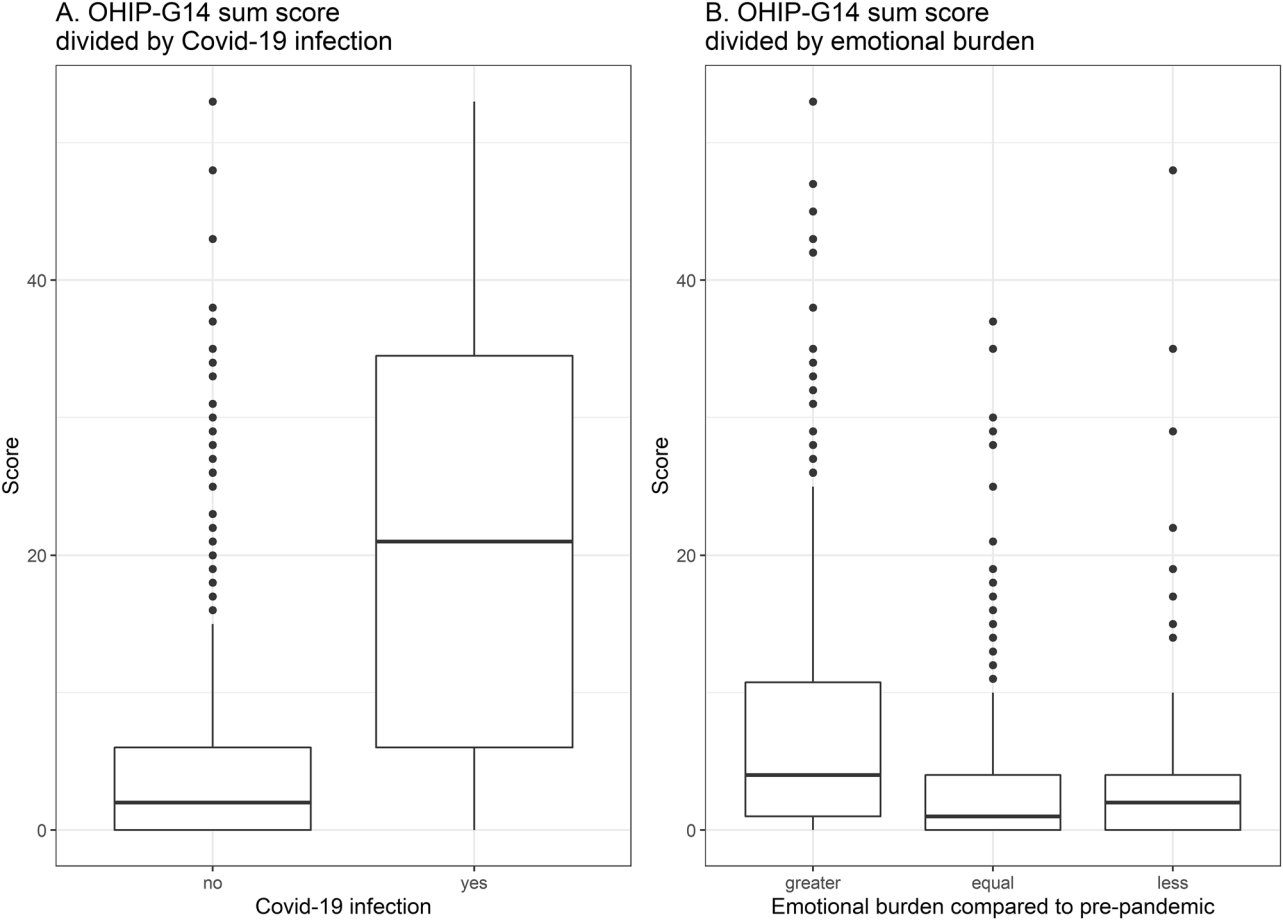
OHIP-G14 sum score stratified to participants with and without COVID-19 history (**A**) and greater, equal or less emotional burden compared to pre-pandemic (**B**).

The variable with the strongest impact on a lower OHIP-G14 sum score is a confirmed COVID-19 history with an estimate of 12.05, meaning that participants with COVID-19 history have on average a 12.05 significantly increased OHIP-G14 sum score compared to participants without COVID-19 history. In addition, an increase of one unit of the PHQ-4 sum score associates with a statistically significant increase of 0.973 in the OHIP-G14 sum score. PHQ-Stress sum score is significantly relevant with an estimate of 0.585, as well as the age group of 60–100 years with an estimate of 1.706 compared to the age groups of 40–59 years (0.848) and 18–39 years (0). Participants with systemic diseases have an increase of 0.89 in the OHIP-G14 sum score compared to participants without systemic diseases. Sex and pandemic related restrictions in life were not statistically significantly relevant in the final model. Figure 1 depicts the differences of the OHIP-G14 sum scores stratified to participants with and without COVID-19 history as well as to participants with greater, equal or less emotional burden compared to pre-pandemic.

## Discussion

In Germany, public and private life has been restricted by lockdown and contact restrictions since March 2020 due to the COVID-19 pandemic. To our knowledge, this is the first paper that uses validated questions to investigate associations of psychosocial factors on oral health and oral afflictions during the COVID-19 pandemic.

In contrast to a low OHIP-G14 sum score of 4.8 ± 7.5 in the overall cohort, suggesting a rather high OHRQoL^17^, the sum scores of participants with self-assessed greater (7.7 ± 9.4), equal (3.1 ± 5.1) and less (3.8 ± 7.1) emotional burden compared to pre-pandemic were statistically significantly different indicating an influential factor of psychosocial factors during the COVID-19 pandemic on OHRQoL. The differentiation of participants with subjectively rated higher, equal, or less emotional burden compared to pre-pandemic is reflected in statically significant group-differences in the more objectified scores of the PHQ-4 and PHQ-Stress sub-questionnaires.

The study design does not allow the detection of causal relationships, but the results might indicate an association between a stated COVID-19 history and increased anxiety and depression level as well as stress on OHRQoL. This can be seen in the linear regression result for modeling the OHIP-G14 score and the variables “PHQ-4” and “PHQ-Stress”. The significant differences in the PHQ-4 sum scores between emotionally greater burdened (4.0 ± 2.8) and equally (1.4 ± 1.7) or less (1.4 ± 1.8) burdened compared to pre-pandemic should be emphasized, since from a sum score of 3.0 onwards a moderate severity of depression and anxiety has to be assumed^19^. The results on stress exposure suggest a mild manifestation in participants with greater emotional burden compared to participants with equal or less emotional burden compared to pre-pandemic and a minimal stress exposure^18^. Explanations could be the altered living conditions for many people with uncertain employment states, financial worries or loneliness^3,20,21^. Brooks et al. showed negative psychological effects of quarantine measures during past pandemics. Stressors included longer quarantine duration, infection fear, frustration, boredom, inadequate supplies, inadequate information, financial loss and stigma. Long-lasting effects are assumed^22^. The COVID-19 pandemic has generally led to short term as well as long term psychosocial and mental health implications^23^. Peters et al. also concluded psychosocial effects of the COVID-19 pandemic from a national cohort study in Germany. In May 2020—at a similar point in time as in this observation—stress was also evaluated with the PHQ-Stress module, depression with PHQ-9 and anxiety with GAD-7. Due to the possibility of comparison with pre-pandemic results, a longitudinal assessment was possible and showed an increase in the PHQ-Stress score of 1.14 ± 0.02, an increase in PHQ-9 by 0.38 ± 0.02 and an increase of the GAD-9 by 0.36 ± 0.02.^24^ Samuel et al. presented similar results, as higher values of the Fear of COVID-19 scale suggest a reduced OHRQoL^25^.

Due to different incidence rates on regional and national levels as well as varying socio-political circumstances and reactions, the effects on social, professional and personal life may be diverse. Therefore, generalization of the present results, but also previous research on this topic, should only be transferred to similar circumstances and conditions. Nonetheless, Kishi et al. also demonstrated disaster-related effects on OHRQoL and mental health following the East Japan Earthquake and tsunami in 2011^26^.

During the COVID-19 pandemic in Germany, dental care was partially limited and essential oral health care and inequities were a matter of debates. The results of the present study underline a continuing need for dental care. 21% of the respondents reported toothache, 23% mucosal problems, 31% hypersensitivity and 27% reported myofacial pain—the same applies to discussions on mental health care^27–30^.

One limitation of this study is that there are no clinical but rather questionnaire-based data for evaluation. However, for the most part, the questionnaire consists of validated items used in epidemiological studies, such as the OHIP-G14, the PHQ-Stress and the PHQ-4 along with the proposed DG PARO periodontitis risk score^16–19^. Furthermore, the known confounders for periodontitis and oral diseases, which can also influence OHRQoL, were integrated^31^.

In addition, a possible Hawthorne effect must be considered, since psychosocial consequences of the COVID-19 pandemic are generally discussed in social discourse^32^. Differences between the two testing formats—digital vs. paper-based questionnaire—have been shown, since the paper-based version was distributed especially to older adults with no access to the internet.

In the study cohort all age groups are represented, the genders and socio-economic status are adequately distributed and people from all federal states took part in this survey. However, due to the localization of the study center and the associated accessibility, many participants are from Baden-Württemberg and Hesse and possibly former patients of the Clinic of Conservative Dentistry at Heidelberg University. Therefore, this study cohort is not representative for the overall German population. This selection bias needs to be taken into consideration, when interpreting e.g. oral hygiene-related parameters. For example, interdental care is rated rather high which is reflected by 43.8% of the study participants using interdental brushes. In contrast, the broad-based and latest Fifth German Oral Health Study from 2013/2014 indicated a prevalence for the usage of interdental brushes of 16.5% for younger adults aged 35–44 years and a prevalence of 29% for younger seniors aged 65–74. Interestingly, in the preceding study of 2005, these values were 10.8% and 14.4%, respectively. This development could indicate that a further increase at an empirical level could be expected through enhanced education in preventive measures in recent years^33^. Thus, the results of this study could either due to the explicit patient collective or follow a positive trend in the usage of interdental brushes in general.

As shown in the regression analysis with variable selection, a COVID-19 history, the PHQ-4 sum score, the PHQ-Stress sum score, age and systemic diseases exerted an influence on OHRQoL. Consensually, the impact of sociodemographic factors on OHRQoL and periodontitis has been described^34,35^.

## Conclusion

Within the limitations of this study, the perception of stress during the first wave of the COVID-19 pandemic in Germany was rated as mild and the severity of depression and anxiety level mild to moderate. A COVID-19 history as well as aggravated level of depression and anxiety and psychological stress were negatively associated with OHRQoL. The depicted oral afflictions underline the continuous need for dental treatment in pandemic times. As the COVID-19 pandemic and its implications progress and in context of future pandemics, psychosocial consequences and their association to oral health should be considered and further investigated.
